# Sleep disruption and adverse pregnancy outcomes

**DOI:** 10.1186/1471-2393-12-S1-A11

**Published:** 2012-08-28

**Authors:** Louise M O’Brien

**Affiliations:** 1 Sleep Disorders Center, University of Michigan, Ann Arbor, MI, USA

## 

A recent meta-analysis reported the most important and potentially modifiable risk factors for stillbirth [1]. Approximately half of these risk factors are likely influenced by maternal sleep disruption. Poor sleep occurs for many reasons although the most common sleep problems include:

• sleep restriction (short sleep duration), which is often self-imposed because of busy lifestyles;

• sleep-disordered breathing (SDB), a term which describes a spectrum of breathing problems during sleep from habitual snoring to obstructive sleep apnea, resulting in oxygen desaturation and sleep fragmentation; and

• poor sleep quality.

Emerging literature now suggests that sleep disruption during pregnancy is associated with poor pregnancy outcomes for both mother and infant [2]. There are emerging associations between maternal sleep and several major risk factors for stillbirth: maternal obesity, gestational hypertension/pre-eclampsia, gestational diabetes, and intra-uterine growth restriction (IUGR).

In recent years, sleep duration has drastically fallen and has been paralleled by a rise in the prevalence of obesity. Chronic sleep restriction (such as self-imposed short sleep duration) plays a pivotal role in the pathophysiology of overweight and obesity via the modulation of neuroendocrine function. Sleep disruption, including short sleep duration and sleep fragmentation, has emerged as a major determinant of metabolic health, independently of weight and is implicated in poor glucose control [3] and possibly gestational diabetes.

Of note, approximately half of all Western women of childbearing age are overweight or obese and obesity can lead to increased risk for sleep disorders such as SDB. Habitual snoring is the main symptom of SDB and its frequency reaches a peak in the third trimester, affecting approximately one third of pregnant women in general, and the majority of those with pre-eclampsia. Habitual snoring is independently associated with gestational hypertension and pre-eclampsia [4]. Although the pathogenesis of pre-eclampsia is not completely understood, the biological pathways include endothelial dysfunction, oxidative stress, and inflammation. The pathogenic process likely originates in the placenta during early pregnancy with abnormal implantation and vasculature development. This leads to oxidative stress and inflammation with subsequent release of anti-angiogenic factors and widespread endothelial dysfunction. Sleep disruption, including poor sleep quality, in early pregnancy has been suggested to adversely impact implantation [5] which has the potential to accelerate the cascade of inflammation and oxidative stress described above. Notably, the mechanisms of sleep disruption that affect cardiovascular morbidity are remarkably similar to the biological pathways for pre-eclampsia.

Poor sleep quality during pregnancy is already evident in the first trimester and has been associated with increased risk for longer labors and Caesarean section delivery [6], as well as preterm delivery [5], likely via its impact on neuroendocrine, metabolic, and inflammatory pathways. In women with SDB, case reports have shown that maternal obstructive apneas are associated with fetal heart rate decelerations, perhaps due to uteroplacental hypoperfusion, a mechanism implicated in intrauterine growth restriction (IUGR).

In summary, maternal sleep disruption is emerging as a significant factor in adverse pregnancy outcomes. Future research should consider maternal sleep when investigating stillbirth.
